# Comparative Study of the Structural, Microstructural, and Mechanical Properties of Geopolymer Pastes Obtained from Ready-to-Use Metakaolin–Quicklime Powders and Classic Geopolymers

**DOI:** 10.3390/ma17164151

**Published:** 2024-08-22

**Authors:** Maroua Zerzouri, Rabah Hamzaoui, Layella Ziyani, Saliha Alehyen

**Affiliations:** 1 Ecole Spéciale des Travaux Publics, Institut de Recherche en Constructibilité, Université Paris-Est, 28 avenue du Président Wilson, 94234 Cachan, France; 2 Ecole Normale Supérieure de Rabat, Laboratoire de Physico-Chimie des Matériaux Inorganiques et Organiques, Centre des Sciences des Matériaux, Université Mohammed V, Avenue Mohamed Bel Hassan El Ouazzani, Takaddoum-Rabat BP 5118, Morocco

**Keywords:** metakaolin, geopolymer, mechanosynthesis, quicklime, compressive strength

## Abstract

This study compares the structural, microstructural, thermal, and mechanical properties of geopolymer pastes (GPs) created through traditional methods and those derived from ready-to-use powders for geopolymer (RUPG) materials. The metakaolin (MK) precursor was activated using a sodium silicate solution or CaO and MOH (where M is Na or K). Various ratios of precursor/activator and Na_2_SiO_3_ or CaO/MOH were tested to determine the optimal combination. For RUPG, the MK precursor was activated by replacing the sodium silicate solution with quicklime. Metakaolin, alkaline hydroxide, and quicklime powders were mixed at different CaO ratios (wt%) and subjected to extensive ball milling to produce RUPG. The RUPG was then hydrated, molded, and cured at 20 °C and 50% relative humidity until testing. Analytical methods were used to characterize the raw and synthesized materials. Classic geopolymers (CGPs) activated with quicklime burst after one hour of molding. The results indicated slight amorphization of GP compared to raw MK, as confirmed by X-ray diffraction analysis, showing N(K)-A-S-H in CGP and N(K)-A-S-H with calcium silicate hydrate (C-S-H/C-A-S-H) in RUPG. The compressive strength of MK-based geopolymers reached 31.45 MPa and 34.92 MPa for GP and CGP, respectively, after 28 days of curing.

## 1. Introduction

Geopolymer is a type of inorganic polymer made from the reaction of an aluminosilicate source material with an alkaline activator solution. Geopolymerization is the process by which these materials are transformed into solid, durable materials with properties similar to traditional cement [1]. 

Geopolymers have several advantages, including high strength and durability, low permeability, and resistance to chemical attack. They also have a lower carbon footprint than cement-based binders, as they require less energy and do not produce as much carbon dioxide during production [2,3,4]. Therefore, they can be used in a variety of applications, including construction materials, coatings, and composites. They have the potential to be a sustainable alternative to traditional cement-based concrete and can help reduce the environmental impact of the construction industry [5]. 

While geopolymer technology offers several advantages over traditional cement-based materials, there are also some limitations to their use, such as the following:

- The complexity of production: The process of elaboration of geopolymer materials can be complex and requires specialized knowledge and equipment [6,7].
- Environmental concerns: While geopolymer materials have lower greenhouse gas emissions than traditional cement-based materials, the production of the alkaline activator solution can still have an environmental impact and can cause significant health risks [6,7].

For these reasons, the use of geopolymers is still limited to prefabricated applications. The best way to achieve the large-scale application of geopolymers is to propose new manufacturing methods. 

Researchers have implemented new ways to produce geopolymers from a ready-to-use precursor powder that can be mixed directly with water like OPC. The first dry mix alkali-activated binder was proposed in 1940 by Purdon et al., who mixed slag and solid sodium hydroxide with water to prepare a mortar mixture [8]. After that (in the 1980s), Heitzmann et al. [9] patented a dry mixture of a binder, composed of different types of aluminosilicate (metakaolin, slag, fly ash, calcined clay), with sources of silicate and potassium hydroxide blended with Portland cement or with a combination of Portland cement and fly ash to provide a curable composition. Kolousek et al. [10] calcined low-grade kaolin together with alkali hydroxides at 550 °C for 4 h and then pulverized them with water to produce one-part geopolymers. However, the one-part geopolymers exhibited extremely low compressive strength. Nematollahi et al. [11,12] developed a sustainable ambient temperature-cured ‘dry-mix’ geopolymer composite. They used a mix of fly ash and two distinct types of slag, which were activated using different solid activators, specifically, different forms of dry sodium silicates in conjunction with sodium hydroxide. To produce the one-part geopolymer matrix, the fly ash, slag, and solid activators—each in powdered form—were first introduced into a Hobart mixer and dry-mixed for approximately three minutes. Following this, water was gradually added to the mixture, and the blending process was continued for an additional eight minutes. 

Among the one-part formulation processes, mechanosynthesis should be mentioned. Mechanosynthesis is defined as a “solventless” method of high-energy ball milling, characterized by the motion of repeated soldering/desoldering of the powder mixture. This motion allows one to obtain metastable crystalline or nanocrystalline phases and convert crystalline phases into amorphous phases and vice versa [13,14]. Mechanosynthesis, or high-energy milling, has been widely used in construction. Nevertheless, it has been tested very little in geopolymer materials. This technique is widely used to increase the reactivity of aluminosilicate precursors and has been effective in improving the mechanical properties of geopolymers obtained from these precursors, even with poor-quality ones [15,16,17]. This would expand the list of aluminosilicate precursors that can be recovered for the production of geopolymer materials. Mechanosynthesis can also reduce the size of the aluminosilicate precursors and combine them with the solid alkaline activators to form precursor powders with geopolymers (pre-geopolymer powders) that are ready to use, storable, easy to transport, and only need to be mixed with water.

Kumar and Kumar [15] studied the effect of the mechanical activation of fly ash on the reaction, structure, and properties of the resulting geopolymer at ambient and elevated (60 °C) temperatures using a isothermal conduction calorimeter. The mechanical activation of fly ash was carried out in laboratory-size eccentric vibrating mills for different milling times (from 5 to 90 min). The authors concluded that under both the conditions (ambient and 60 °C), mechanical activation enhanced the rate and decreased the time of reaction. Mucsi et al. [16] compared different milling techniques (drum-ball, vibratory, and agitated mills) to improve the fly ash’s reactivity; they were used as precursors for the production of geopolymers. The use of the agitator mill resulted in an elevated specific surface area of fly ash and the increase in geopolymer compressive strength, compared to the binders elaborated with the unmilled fly ash. Xia and Sanjayan [17] milled activators (anhydrous sodium and metasilicate beads) in a planetary ball mill for 5 min before mixing them with a slag aluminosilicate source and fine sand in a Hobart mixer to obtain a homogeneous concrete mixture. The compressive strength reached 16 MPa after 7 days of curing. Zerzouri et al. [6,7] studied the feasibility of preparing a geopolymer precursor powder (pre-geopolymer powder (PGP)) using the mechanosynthesis process. A dry mixture of aluminosilicate source (fly ash or blast furnace slag) and alkaline activators with different Na_2_SiO_3_/NaOH mass ratios were milled together using planetary ball milling. The results showed that hydrated PGP binders present a similar structure to the classic geopolymers. Testing of mechanical properties showed that mechanosynthesis could improve flexural and compressive strength [6].

Metakaolin is a natural material that is rich in silica and alumina. It is obtained by the calcination of crude kaolin, which contains kaolinite as the main mineral [18,19,20]. Metakaolin has both an amorphous character, identified in X-ray diffraction by the presence of a centered halo, and a crystalline character, depicted by the presence of crystalline phases such as quartz, anatase, muscovite, kaolinite, etc. It is a material with a high reactivity: its reaction with alkaline solutions leads to the formation of a geopolymer with high mechanical performance [18,19,20]. Metakaolin stands out as the most frequently employed aluminosilicate precursor for geopolymer materials in the existing literature. The primary challenge associated with the use of metakaolin aluminosilicate is related to its cost, which come from its distinct manufacturing process compared to by-products like fly ash [21,22]. In this research, we addressed the cost challenge by utilizing flash-calcined metakaolin. R. San Nicolas et al. [22] conducted a study comparing traditional calcined metakaolin with flash-calcined metakaolin, demonstrating that the flash calcination process induces certain physical modifications and imparts a specific composition, resulting in a relatively reasonable price [21,22]. 

In the geopolymerization reaction, sodium/potassium hydroxide is considered as the primary activator by providing the alkalinity needed for the initiation of the geopolymerization reaction. Sodium silicate, on the other hand, supports the reaction by supplying additional silica and alumina ions, contributing to the overall formation and stability of the geopolymer structure [23,24]. 

The use of CaO as activator was proved by many researchers to be advantageous for geopolymers with a room temperature curing condition and has an especially significant influence on the mechanical properties of metakaolin-based geopolymers. The added CaO can react with the silica (in the metakaolin) to form C-A-S-H/C-S-H hydrates. The formation of such hydrates might cause a water reduction in the alkaline medium, resulting in a higher alkalinity medium, therefore enhancing the dissolution of the Si and Al species [25,26].

In this study, the mechanosynthesis technique was employed to produce ready-to-use metakaolin-based powders that are safe for health and easy to store and transport. This work aims to elaborate ready-to-use powders for geopolymers (RUPGs) on metakaolin, activated by a mixture of alkaline hydroxides and quicklime and to investigate their physico-chemical and mechanical properties before and after hydration.

## 2. Materials and Methods

### 2.1. Materials

The metakaolin (MK) used in this work came from Argeco® (Argeco Développement Usine de production Rue Fournie Gorre 47500 FUMEL, France). It was obtained through the flash calcination of kaolin. Argeco metakaolin is characterized by its pinkish color and its high content of SiO_2_ and Al_2_O_3_ (Table 1).

Two types of alkali hydroxide were selected: sodium hydroxide in the form of microbeads with a purity of 99%, distributed by Asserdis(39 bis rue du Moulin Rouge 10150, Charmont-sous-Barbuise, France), and potassium hydroxide obtained from Sigma Aldrich (Sigma Aldrich Chimie 80 Rue de Luzais, 38070 Saint-Quentin-Fallavier, France) in the form of white pellets with a purity of 99%.

Sodium metasilicate (meta-Si) microbeads with about 40.3% SiO_2_ and 56.10% Na_2_O and sodium silicate solution with about 68.27% SiO_2_ and 28.20% Na_2_O were distributed by Asserdis.

Quicklime was used instead of alkaline silicates. It was supplied by Asserdis and is characterized by a beige color and a purity of 93%.

### 2.2. Methods

In this study, a Retsch PM 400 planetary ball mill (Retsch France Verder S.A.R.L.8 Allée Rosa Luxembourg Immeuble Arizona 95610 Eragny sur Oise France) with 500 mL steel jars was utilized. To prepare RUPG samples, 250 g of powder was added to each jar, resulting in a ball-to-powder weight ratio of approximately 3.6, selected based on previous research for optimal results with the specific material used. This setup aimed to achieve a uniform and fine powder suitable for subsequent analysis.

Sodium metasilicate caused pre-geopolymer powders to stick in the jars after 3 min of grinding, leading to the replacement of metasilicate with quicklime, in combination with alkaline hydroxide as activators. Two types of alkaline hydroxide, KOH and NaOH, were tested, along with three CaO/MOH ratios (M = K or Na) to find the optimal formula.

**Figure 1:**
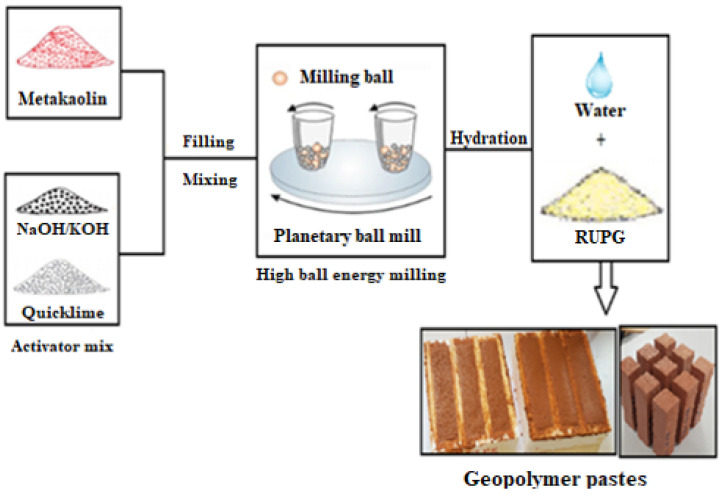
Methodology of making RUPG.

To obtain RUPG (Figure 1), the metakaolin (MK) precursor was mixed with solid-state alkaline activator and quicklime in jars, following the parameters listed in Table 2. The mixture was then milled to produce RUPG. The mass ratio R1 of MK/alkali-activating mix (AA) was kept constant at 4, while the mass ratio R2 of CaO/NaOH or CaO/KOH varied from 0.5 to 1.5. Milling conditions were set to 400 rpm for 3 min. Samples were labeled according to the following convention: RUPG-MK-CN (CaO/NaOH); CK (CaO/KOH); R2 (ratio): Milling time (Mt); Milling speed (Ms).

To prepare the geopolymer pastes, the synthesized RUPG was hydrated with a fixed water-to-solid ratio of 0.25 (wt), determined after conducting several water demand tests. The resulting geopolymer paste (GP) was molded into rectangular polystyrene molds measuring 4 cm × 4 cm × 16 cm. The paste was cured at 20 °C and 50% relative humidity until the day of testing. Physicochemical properties were evaluated after 7 days of curing, while mechanical properties were assessed after 7 and 28 days of curing.

**Figure 2:**
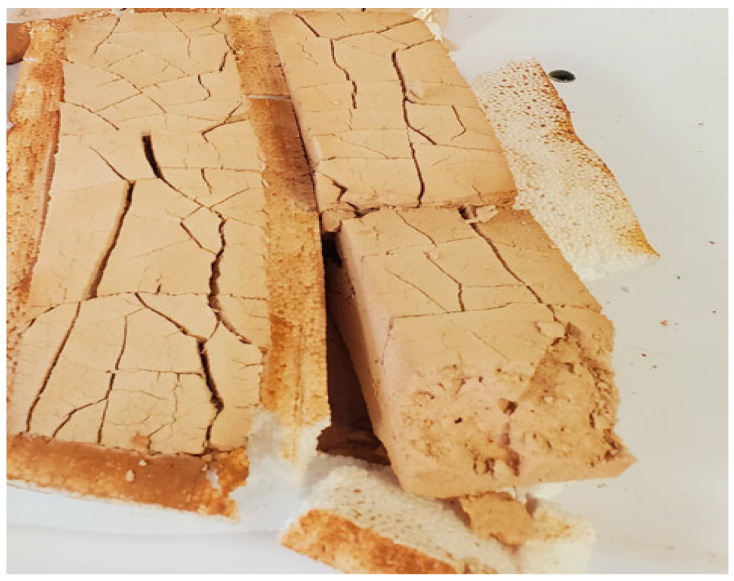
Images of traditional geopolymer pastes synthesized from metakaolin, activated by quicklime and sodium hydroxide (NaOH), showing surface morphology and texture.

The paste produced using the classic method with quicklime exhibited immediate swelling starting from the initial time point (t0) and continued to expand significantly during the first few minutes of drying. This behavior was observed consistently across all tested ratios, as illustrated in Figure 2. Therefore, classic geopolymers based on MK with different ratios of sodium silicate solution (R1 = Na_2_SiO_3_/(NaOH or KOH) (2.5 and 3.5) and R2 = Metakaolin/Alkaline activator (2.5 and 3.5)) were made, labeled as CGP-MK-R1-R2.

Various analytical methods were employed to characterize both the raw and synthesized materials. The geopolymer pastes were analyzed after being crushed at 7 days of curing. Pieces of the geopolymer materials were first crushed and then sieved to a particle size of 200 µm prior to analysis.

The particle size distribution was determined using a laser particle analyzer (LS 13 320 XR, Beckman Coulter). The chemical composition was analyzed by X-ray fluorescence spectrometry using an S2 Ranger model instrument (Bruker) in pellet mode.

To determine the mineralogy, X-ray diffraction (XRD) was utilized with a D2 Phaser diffractometer (Bruker) equipped with a Cu-Kα copper X-ray tube (λ = 1.54 Å). The samples were scanned at an angular range between 5 and 60° (2θ), with a step size of 0.02 and a time interval of 0.1 s. The software DIFFRAC.EVATM, including the ICDD PDF4 database, was used for analysis. The Rietveld method was used to quantify the mineral phases identified by XRD and the sample’s amorphization rate, using Bruker’s Topas V6 software.

Thermal characteristics were assessed using TG analysis with a TGA/DSC 2 instrument (Mettler–Toledo) at a heating rate of 20 °C/min, ranging from 25 °C to 1000 °C, with nitrogen as the purge gas at a flow rate of 40 mL/min.

Chemical bonds were determined using Attenuated Total Reflectance Fourier Transform Infrared (ATR-FTIR) spectrometry with a Spectrum Two instrument (Perkin Elmer). Powders or crushed solid pastes were placed on a diamond crystal for analysis, and spectra were collected in the range of 4000–400 cm^−1^, with 4 cm^−1^ resolution and 64 scans.

The compressive strength of the geopolymer pastes was measured using a Syntax electromechanical press (3R) with a maximum force of 300 kN after 7 and 28 days of curing. Scanning electron microscopy observations were conducted in the laboratory of Sorbonne University, UPMC campus, using a Gemini microscope model SUPRA 55VP (Zeiss) coupled to an energy-dispersive analysis probe (EDS).

## 3. Results and Discussions

### 3.1. Ready to Use Powders

**Figure 3:**
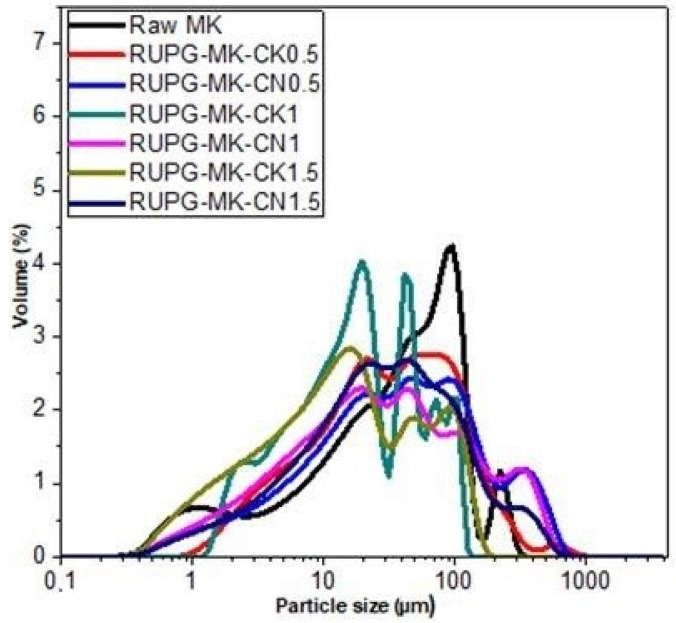
Particle size distribution of raw MK and RUPG obtained at 400 rpm and 3 min of milling.

The particle size distributions of the raw metakaolin (MK) and MK-RUPG powders are presented in Figure 3. Raw MK exhibits a wide particle size distribution ranging from 0.4 to 310 µm. During the milling process of the different MK-based RUPG samples, new peaks appear between 20 and 70 µm, which are attributed to the high-energy milling process, allowing the mixture of metakaolin with the alkaline activators. This phenomenon is possibly due to the agglomeration of particles, which is related to the cold-welding effect. These findings are consistent with the results obtained by Bouchenafa et al. [27], who performed high-energy milling of fly ash using a planetary ball mill. The authors pointed out the emergence of a new zone after 15 min of milling and explained it as the agglomeration of fine particles during the milling process.

Generally, the effect of the mechanosynthesis process on particle performance can be summarized in three stages. In the first stage, particles rearrange and stack, sliding past each other with minimal deformation and fracture, resulting in a reduction in particle size and shape modification. In the second stage, particles undergo elastic and plastic deformations, and the cold-welding phenomenon is observed, resulting in agglomeration and an increase in the particle size of the powder. In the last stage, particles are fractured, leading to further deformation and fragmentation and ultimately reducing the particle size [27,28,29]. It is important to note that the milling process steps depend directly on the size of the initial powders. If the particle size is less than 5 µm, the grinding process can initially promote agglomeration, resulting in the formation of coarse particles that can undergo the necessary deformations for the take-off phenomenon. Typically, the finished product consists of a combination of fine, medium, and coarse particles, where further grinding has no significant effect on the particle size. At this stage, a stationary state of milling is reached [27,28,29]. In this study, planetary ball mills were used, and depending on the milling conditions and the elements placed in the vials, three distinct motion types of the milling balls were characterized: cascading, cataracting, and rolling. In the cascading regime, the milling balls are carried along by the vial’s wall and tumble over each other, moving from the top of the bulk to its base. For the cataracting regime, balls detaching from the wall and impact the treated material or the opposite wall with high intensity. The rolling regime or centrifuging balls are aligned to the wall rotation with almost no relative velocity, and milling thus becomes less efficient [30].

**Figure 4:**
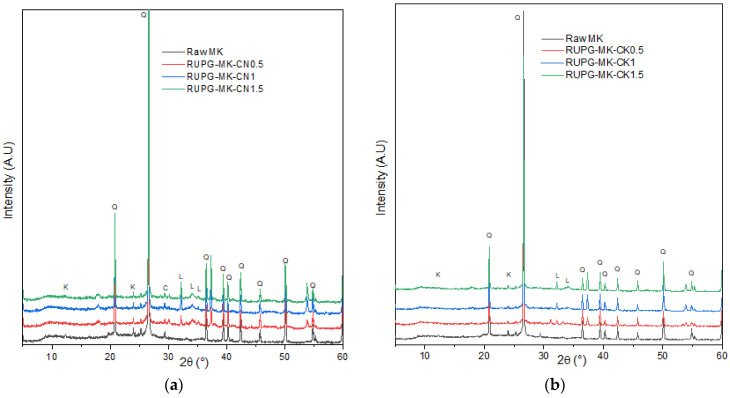
XRD diffractograms of raw metakaolin and RUPG activated with (**a**) NaOH and (**b**) KOH. Q: Quartz (PDF 00-005-0490), C: Calcite (PDF 00-003-0526), K: Kaolinite (PDF 01-078-2110), and L: Lime (PDF 00-002-1088).

**Figure 5:**
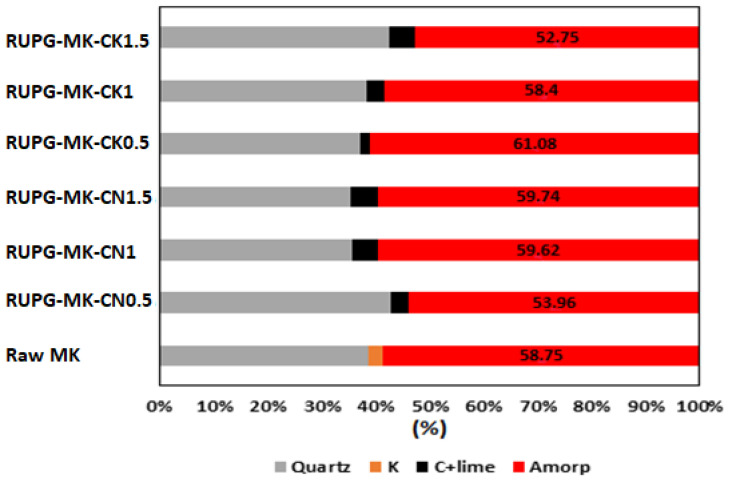
Quantification of crystalline phases and amorphous content by the Rietveld method for MK and RUPG. Q: quartz (PDF 00-005-0490), C: calcite (PDF 00-003-0526), K: kaolinite (PDF 01-078-2110), Amorp: amorphous rate.

In Figure 4, the X-ray diffraction (XRD) pattern of both raw metakaolin (MK) and RUPG are presented. Raw MK mainly consists of quartz and some traces of kaolinite. In the RUPG, we observe a slight amorphization, indicated by a decrease in intensities and broadening of the quartz peaks, which is attributed to the effect of mechanosynthesis [27,31]. The amorphization is further confirmed through quantification using the Rietveld method (Figure 5). The sample with a CaO/KOH ratio of 0.5 exhibits an optimum amorphization rate of 61.08%.

**Figure 6:**
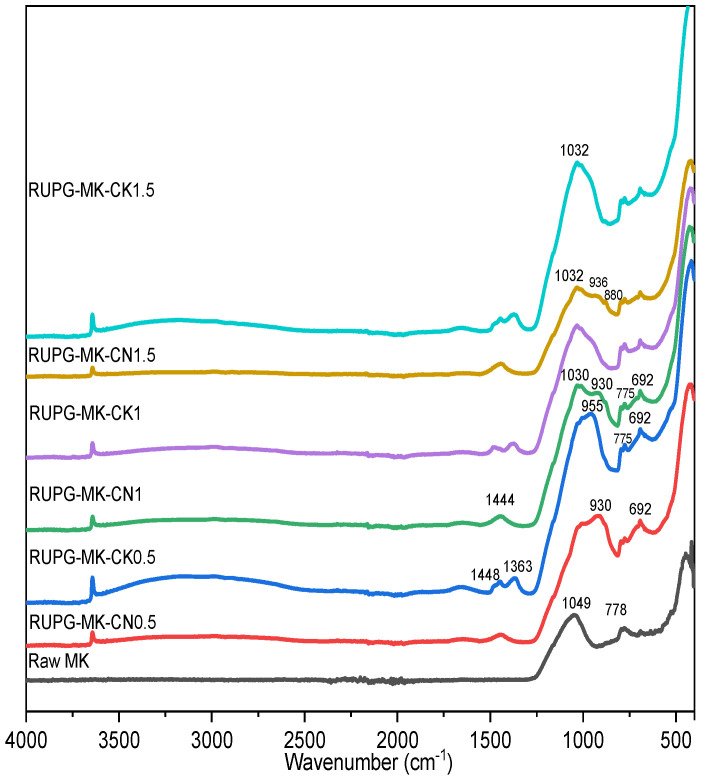
Infrared spectra of raw MK and RUPG.

The infrared spectra analysis of metakaolin (depicted in Figure 6) reveals three main bands at 1049, 778, and 692 cm^−1^. The band located at 1049 cm^−1^ corresponds to the asymmetric stretching vibration of Si-O-T bonds, where T stands for Si or Al [32]. The two bands observed at 778 and 692 cm^−1^ are associated with the deformation vibrations of the Si-O bonds from quartz [33].

Remarkably, all pre-geopolymer powders show a shift in the band related to asymmetric stretching vibration of Si-O-T (1049 cm^−1^) bonds toward lower wavenumbers, leading to the emergence of the bands around 1030–1032 cm^−1^ that are commonly reported as characteristic bands of Si-O asymmetric stretching vibrations [34]. This shift decreases with an increase in the CaO/(NaOH or KOH) ratio, reflecting the decrease in the alkalinity of the activator mixture [35].

Furthermore, the bands observed around 930 cm^−1^ indicate the presence of Si-O-Ca bond vibrations [36]. The interval of 1350–1450 cm^−1^ contains bands that can be attributed to the vibration modes of the C–O group, resulting from the carbonation reaction between the unreacted CaO and the CO_2_ in the atmosphere [37,38]. Additionally, the signals present in the 692–702 cm^−1^ region may be attributed to Si-O-Si vibrations [34].

### 3.2. Comparative Study of the Properties of Geopolymer Pastes Produced from RUPG and Classic Geopolymers

**Figure 7:**
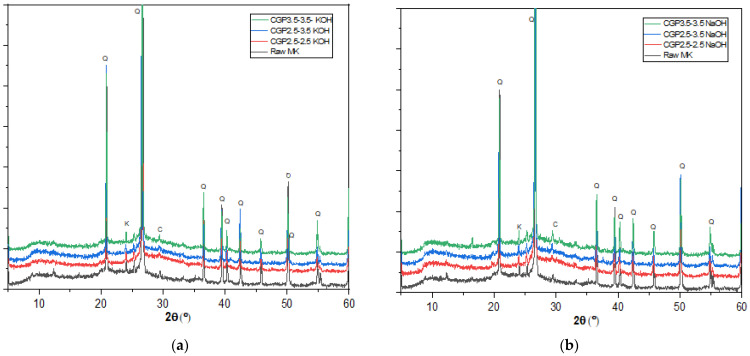
XRD diffractograms of raw metakaolin and Classis geopolymer pastes with (**a**) KOH and (**b**) NaOH (K: Kaolinite (PDF 01-078-2110), C: calcite (PDF 00-003-0526), Q: Quartz (PDF 00-005-0490)).

XRD patterns of geopolymer pastes manufactured using the classic method (Figure 7) show a shift in the halo observed in the raw MK between (20° and 30°) to higher 2Ɵ value (25° to 35°) whatever the activator nature (KOH Figure 7a, or NaOH Figure 7b). This halo shift is characteristic of the formation N(K)-A-S-H geopolymer product [39,40]. 

**Figure 8:**
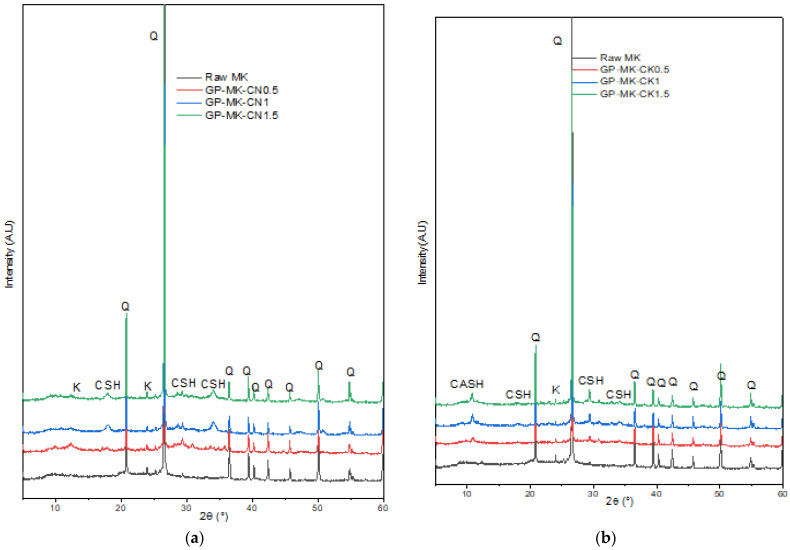
XRD diffractograms of raw metakaolin and geopolymer pastes based on RUPG activated with (**a**) NaOH and (**b**) KOH (K: kaolinite (PDF 01-078-2110), Q: quartz (PDF 00-005-0490), CSH: calcium silicate hydrate (PDF 00-003-0548), CASH: calcium aluminum silicate hydrate (PDF 00-015-0171)).

XRD patterns in Figure 8 showed that the produced geopolymer pastes from RUPG kept the same structure as the raw MK. We note the appearance of C-S-H and C-A-S-H, following the alkaline activation reaction with quicklime. The C-S-H peaks are more visible with higher CaO/MOH ratios. Samples with KOH show the appearance of C-A-S-H as well. A decrease in the peak’s intensities corresponding to quartz is also noted; this indicates an amorphization under the effect of milling.

**Figure 9:**
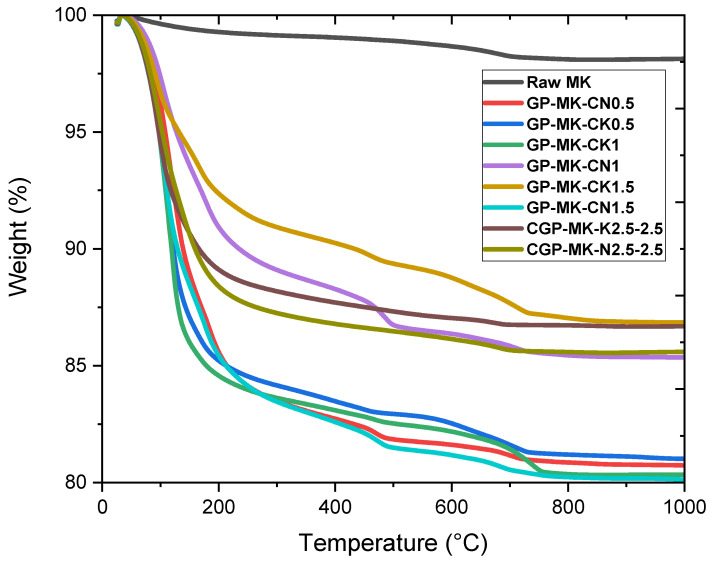
Thermograms of raw metakaolin, geopolymer pastes based on RUPG, and classic geopolymer pastes.

Figure 9 shows TGA curves of raw MK and geopolymer pastes. Raw MK (Figure 9) presents two identified minor mass loss areas, the first one between 50 and 100 °C and the second one between 550° and 680 °C, corresponding to the evaporation of water and the dihydroxylation of the residual kaolinite present in the MK (as shown by the XRD analysis), respectively [20,41,42].

In all the samples of geopolymer pastes, the predominant weight loss was observed at temperatures below 200 °C across all specimens. This phenomenon primarily resulted from the swift migration of interstitial water towards the surface, followed by its subsequent evaporation [43]. Moreover, a progressive reduction in mass (approximately 1 to 2%) is observed at a slow rate between 350° and 650 °C. This phenomenon is likely associated with the dihydroxylation process and the establishment of new T-O-T linkages, contributing to the compaction of all matrices [43]. During this phase, the minor mass loss is probably attributed to the evaporation of the chemically bonded water, the degradation of the N(K)-A-S-H gel, and hydroxyl groups in the geopolymer matrix. [43,44,45].

Geopolymer pastes derived from RUPG using a NaOH activator exhibit an additional mass loss within the range of 180–250 °C, a characteristic commonly associated with C-S-H dehydration, as documented in the literature [46,47,48,49]. Conversely, geopolymer pastes from RUPG with KOH display a more substantial mass loss at 110 °C, potentially attributable to the presence of C-A-S-H in this context [50,51]. 

**Figure 10:**
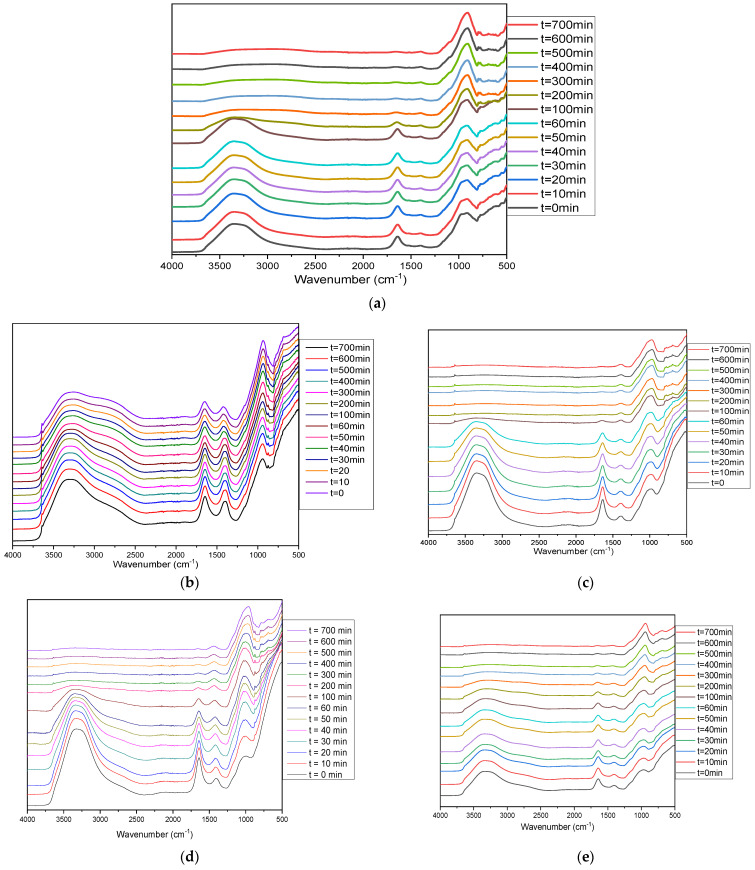

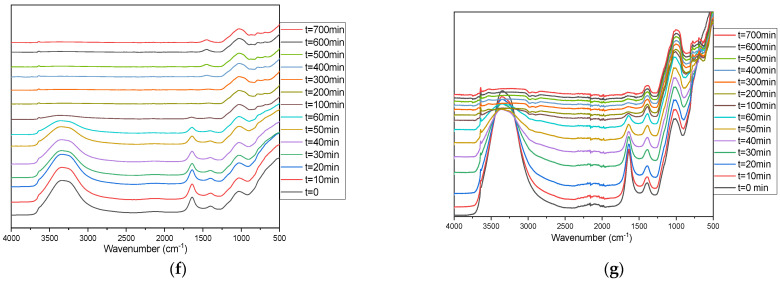
In situ ATR-FTIR spectra of (**a**) CGP-MK-K2.5-3.5, (**b**) GP-MK-CN0.5, (**c**) GP-MK-CK0.5, (**d**) GP-MK-CN1, (**e**) GP-MK-CK1, (**f**) GP-MK-CN1.5, (**g**) GP-MK-CK1.5.

**Figure 11:**
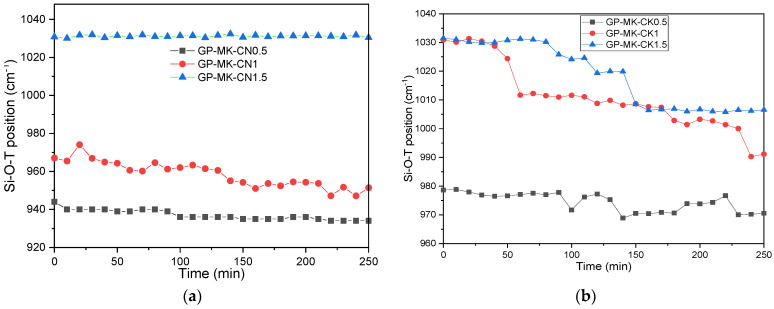
Shift in the Si-O-T band position from IR spectra versus time for hydrated RUPG activated with NaOH (**a**) and KOH (**b**).

As noticed in the pre-geopolymer powder spectra, the main band of raw MK located around 1049 cm^−1^ moved towards lower wavenumbers. For the classic geopolymer (Figure 10a), the main band moved from 1049 cm^−1^ to 930 cm^−1^ after 13 h of geopolymerization reaction. After hydration (Figure 10 and Figure 11a,b), this band continues to move toward lower wavenumbers. This characteristic shift of the polycondensation reaction, accompanied by a decrease in the intensity of the bands characteristic of the H-O-H groups observed at 1640–1560 cm^−1^ and of the Si-O-H groups at 3200 cm^−1^, is representative of the formation of a geopolymer network. The displacement is less important for samples with a high CaO content. The GP-MK-CK1.5 sample (Figure 11b) shows a different behavior. The main MK band moves from 1049 cm^−1^ to 1019 cm^−1^, then moves in the opposite direction starting at 60 min and stabilizes around 1030 cm^−1^. This well-known band characterizes the C-S-H entities [52]. GP-MK-CK0.5 sample shows the largest displacement in the series of MK and CaO samples, with a maximum displacement of 1049 to 960 cm^−1^. We also note the appearance of bands relating to carbonates in the 1380–1430 cm^−1^ region, corresponding to O-C-O [36,53].

This result agrees well with the one found by the XRD technique, indicating the coexistence of geopolymer network and C-S-H/C-A-S-H networks. Yip et al. [54] were the first to consider the coexistence of the compounds C-A-S-H and N-A-S-H, by conducting an alkali-activation study of metakaolin and blast-furnace slag. The authors concluded that the simultaneous formation of C-A S-H and N-A-S-H helps to bridge the gaps between different phases and unreacted particles, resulting in better mechanical performance [54]. The main observed bands are summarized in Table 3.

**Figure 12:**
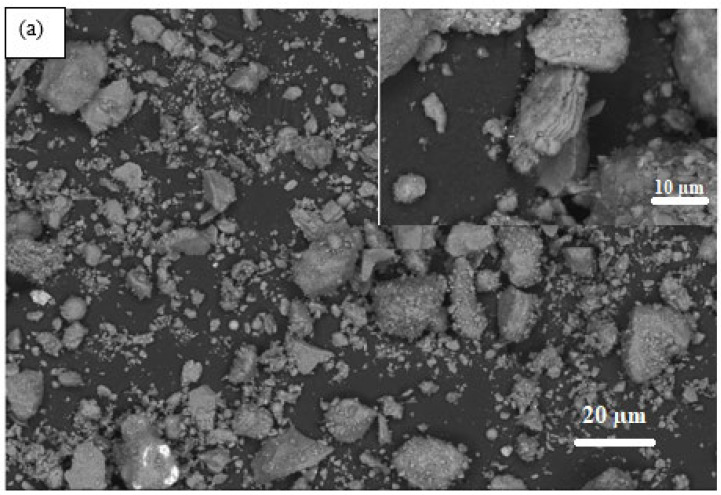

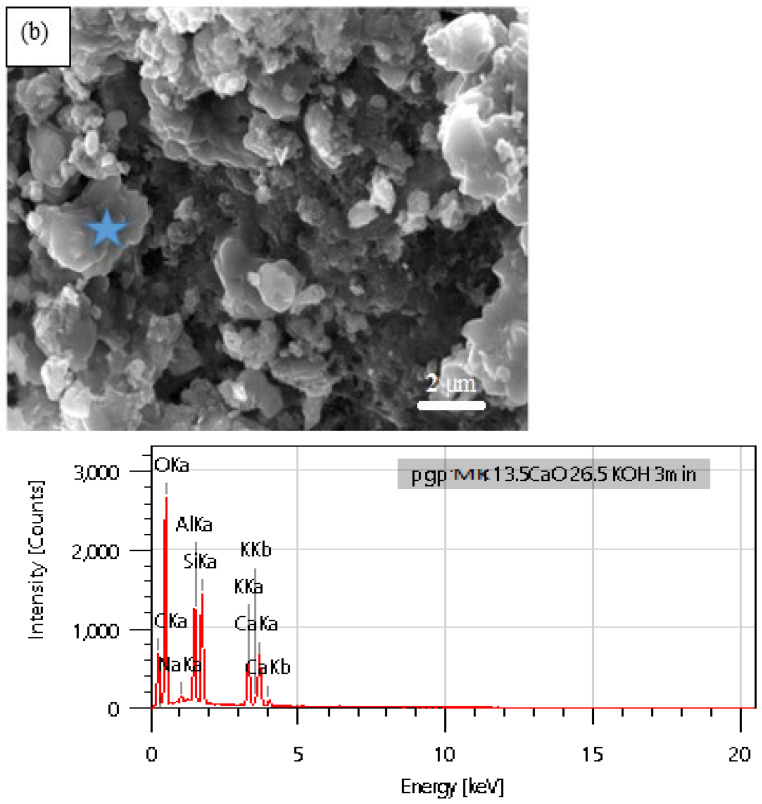

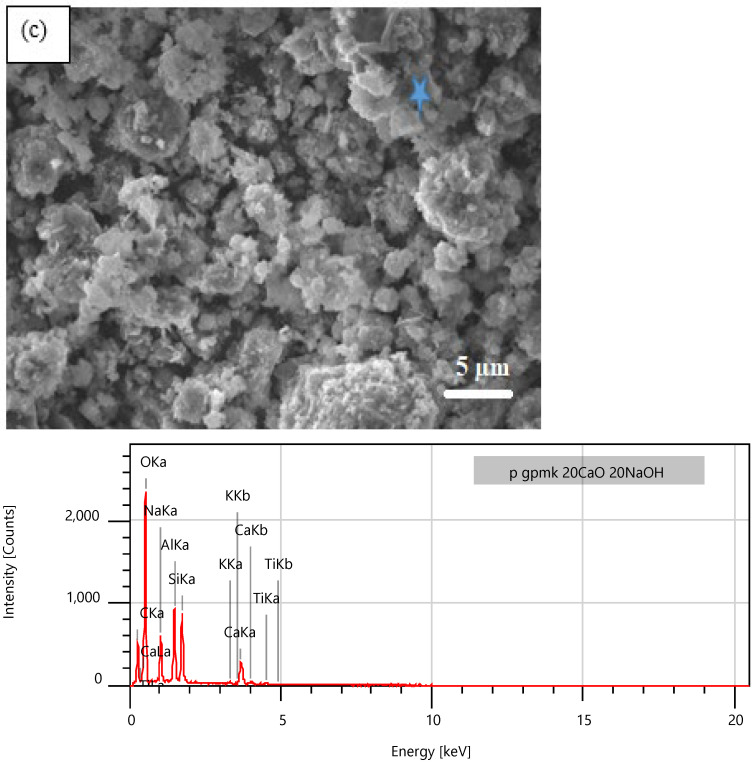

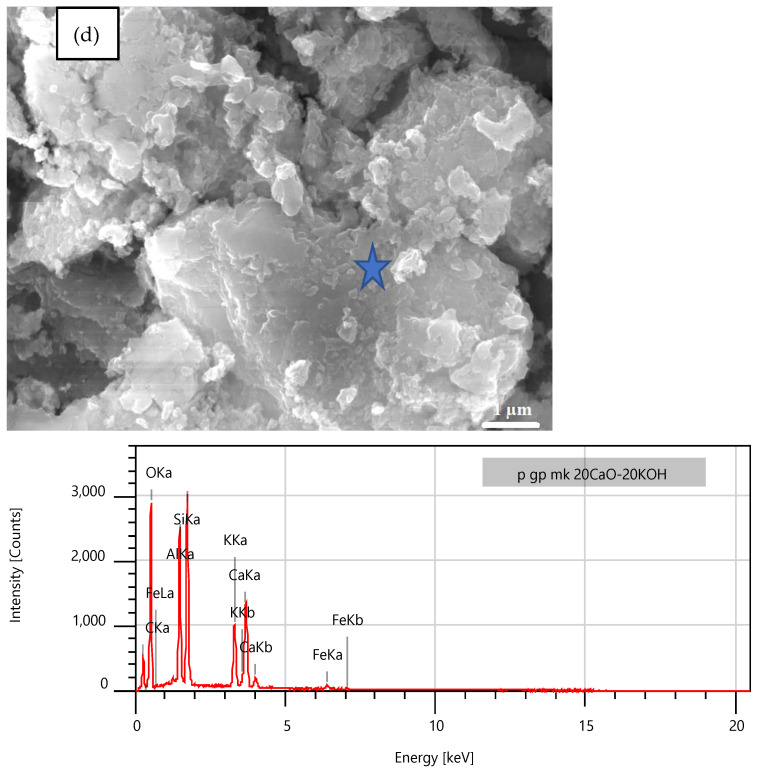
SEM micrographs with EDS analysis of (**a**) raw MK, (**b**) GP-MK-CK0.5, (**c**) GP-MK-CN1, and (**d**) GP-MK-CK1.

Observation of raw MK (Figure 12a) by electron microscopy reveals a fairly diversified morphology that is very heterogeneous and mostly small in size. With a higher magnification, a microstructure is observed in the form of sheets glued together. These observations are in agreement with what is reported in the literature on the microstructure of MK [55].

MK-based geopolymer pastes show the appearance of a continuous dense structure characteristic of geopolymer gels. GP-MK-CK0.5 sample (Figure 12b) shows in EDS a large percentage of the K element, thus indicating the formation of a K-A-S-H network characteristic of potassium-based geopolymers. A ratio of (Ca/Si) of 0.8 with a low presence of Al, which can be attributed to the existence of C-S-H in low quantity, was observed by XRD, confirming peaks of small C-S-H intensities [46,54]. In addition it is found that the GP-MK-CN1 sample (Figure 12c) shows a significant level of Na and Al and traces of Ca, thus confirming the creation of a N-A-S-H network in parallel with C-S-H. Sample GP-MK-CK1 (Figure 12d) shows high levels of Si, Al, Ca, and K, indicating the presence of C-A-S-H in parallel of K-A-S-H, as observed by XRD.

**Figure 13:**
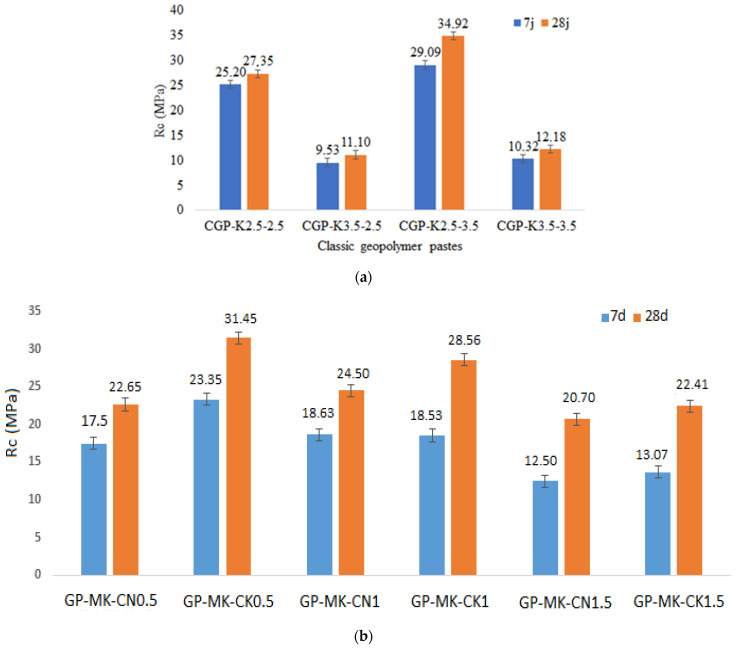
Compressive strength (Rc) after 7 and 28 days of curing for (**a**) classic geopolymer pastes, and (**b**) geopolymer pastes from RUPG.

Figure 13 shows the compressive strengths of the different samples. The KOH-based samples resulted in higher mechanical strengths. This agrees with what has been reported in the literature by several scientific researchers [20,56,57]. The best mechanical performance for RUPG-based pastes (Figure 13a) was recorded for GP-MK-CK0.5 and GP-MK-CN1 samples, with values of 31.45 and 24.5 MPa, respectively, for the two types of activators. Classic geopolymer pastes (Figure 13b) show the best compressive strength for the sample CGP-K2.5-3.5 of about 34.39, which is close to the results found with pastes based on RUPG.

The particle size of the precursor powder plays a significant role in these outcomes. Finer particles increase the surface area, enhancing the reactivity and degree of geopolymerization, which in turn improves the mechanical properties. The in situ IRTF monitoring results revealed the coexistence of two distinct networks: an M-rich network (N-A-S-H or K-A-S-H) and a Ca-rich network (C-S-H and/or C-A-S-H). This was evidenced by the shifting of the characteristic Si-O-T (T=Si, Al) band toward lower, then higher wavenumbers. Notably, the GP-MK-CK0.5 and GP-MK-CK1 samples exhibited the most significant shifts toward lower wavenumbers, indicating a higher degree of geopolymerization. This enhanced geopolymerization, facilitated by the fine particle size, likely explains the superior mechanical resistance observed in these samples, as the high N-A-S-H content contributes to improved mechanical strength over time [6,7].

## 4. Conclusions

This study aimed to evaluate and compare traditional and mechanosynthesis methods of geopolymerization, focusing on the properties of geopolymers produced using metakaolin (MK) activated by different reagents. Specifically, we sought to assess the effectiveness of traditional methods involving sodium silicate or calcium-based hydroxides (CaO/MOH) against a new approach using ready-to-use powders for geopolymers (RUPGs), which utilize mechanosynthesis with quicklime as a substitute for sodium silicate.

Our findings indicate that traditional geopolymerization methods yield geopolymer pastes (GP) with significant properties. The RUPG method, which simplifies preparation and enhances storage convenience, presents a promising alternative. This approach demonstrates the potential for improved practicality in industrial applications.

- Structural and microstructural insights: X-ray diffraction (XRD) analysis reveals that traditional methods produce slight amorphization in MK, resulting in N(K)-A-S-H gels. Conversely, RUPG incorporates additional calcium silicate hydrate (C-S-H/C-A-S-H) phases, suggesting a potentially more robust microstructure that could contribute to enhanced mechanical properties and durability.
- Mechanical properties: Both GP and classic geopolymer pastes (CGPs) achieved notable compressive strengths of 31.45 MPa and 34.92 MPa, respectively, after 28 days of curing. The slightly superior compressive strength of CGP underscores the potential advantages of traditional methods in applications where high strength is crucial.

The study also highlighted challenges such as swelling and expansion during the initial curing of CGP, particularly with quicklime activation. Despite these challenges, the RUPG method offers a safer and more manageable approach with potential for broader industrial application.

## Figures and Tables

**Table 1:** Chemical composition of the used raw MK.

	SiO_2_ (%)	Al_2_O_3_ (%)	Fe_2_O_3_ (%)	CaO (%)	SO_3_ (%)	K_2_O (%)	TiO_2_ (%)	P_2_O_5_ (%)	SiO_2_/Al_2_O_3_ (%)
MK	62.62	29.03	3.19	1.44	0.43	0.50	1.65	0.64	2.16

**Table 2:** Mix design and milling parameters.

Sample ID	R1	CaO/MOH	Mt (min)	Ball/Powder	Ms (rpm)
RUPG-MK-CM1.5	4	1.5	3	4	400
RUPG-MK-CM1	4	1	3	4	400
RUPG-MK-CM0.5	4	0.5	3	4	400

M = K or Na.

**Table 3:** Infrared spectroscopy bands summary.

Wavenumber (cm^−1^)	Attribution
1350–1450	C-O group vibration
1049	Si-O-T (T = Si. Al) asymmetric stretching vibration
1030	Si-O asymmetric stretching vibration
930	Si-O-Ca bond vibration
778 and 692	Si-O bond strain vibration of quartz
692–702	Si-O-Si vibration

## Data Availability

No new data were created or analyzed in this study. Data sharing is not applicable to this article.

## References

[1] Davidovits J. (1991). Geopolymers—Inorganic Polymeric New Materials. J. Therm. Anal..

[2] Singh N.B., Middendorf B. (2020). Geopolymers as an Alternative to Portland Cement: An Overview. Constr. Build. Mater..

[3] Davidovits J. Properties of Geopolymer Cements. Proceedings of the First International Conference on Alkaline Cements and Concretes.

[4] Meesala C.R., Verma N.K., Kumar S. (2020). Critical Review on Fly-Ash Based Geopolymer Concrete. Struct. Concr..

[5] Temuujin J., Rickard W., Lee M., Van Riessen A. (2011). Preparation and Thermal Properties of Fire Resistant Metakaolin-Based Geopolymer-Type Coatings. J. Non-Cryst. Solids.

[6] Zerzouri M., Bouchenafa O., Hamzaoui R., Ziyani L., Alehyen S. (2021). Physico-Chemical and Mechanical Properties of Fly Ash Based-Geopolymer Pastes Produced from Pre-Geopolymer Powders Obtained by Mechanosynthesis. Constr. Build. Mater..

[7] Zerzouri M., Hamzaoui R., Ziyani L., Alehyen S. (2022). Influence of Slag Based Pre-Geopolymer Powders Obtained by Mechanosynthesis on Structure, Microstructure and Mechanical Performance of Geopolymer Pastes. Constr. Build. Mater..

[8] Luukkonen T., Abdollahnejad Z., Yliniemi J., Kinnunen P., Illikainen M. (2018). One-Part Alkali-Activated Materials: A Review. Cem. Concr. Res..

[9] Fitzgerald M., James L. (1987). United States Patent (19). https://patentimages.storage.googleapis.com/eb/9e/dc/50e6b2c00604f0/US4767095.pdf.

[10] Koloušek D., Brus J., Urbanova M., Andertova J., Hulinsky V., Vorel J. (2007). Preparation, Structure and Hydrothermal Stability of Alternative (Sodium Silicate-Free) Geopolymers. J. Mater. Sci..

[11] Nematollahi B., Sanjayan J., Shaikh F.U.A. (2015). Synthesis of heat and ambient cured one-part geopolymer mixes with different grades of sodium silicate. Ceram. Int..

[12] Nematollahi B., Sanjayan J., Qiu J., Yang E. (2017). Micromechanics-Based Investigation of a Sustainable Ambient Temperature Cured One-Part Strain Hardening Geopolymer Composite. Constr. Build. Mater..

[13] Suryanarayana C. (2004). Mechanical Alloying and Milling.

[14] Suryanarayana C. (2001). Mechanical Alloying and Milling. Prog. Mater. Sci..

[15] Kumar S., Kumar R. (2011). Mechanical Activation of Fly Ash: Effect on Reaction, Structure and Properties of Resulting Geopolymer. Ceram. Int..

[16] Mucsi G., Kumar S., Csoke B., Kumar R., Molnár Z., Rácz Á., Mádai F., Debreczeni Á. (2015). Control of Geopolymer Properties by Grinding of Land Filled Fly Ash. Int. J. Miner. Process..

[17] Xia M., Sanjayan J. (2016). Method of Formulating Geopolymer for 3D Printing for Construction Applications. JMADE.

[18] Garcia-diaz E., Réactivité E.G., Garcia-diaz E., Kaolinites M.D.E.S. (2013). Réactivité Pouzzolanique Des Métakaolinites: Corrélations Avec Les Caractéristiques Minéralo-Gitologiques Des Kaolinites. https://theses.hal.science/tel-00843099v1/document.

[19] Wan Q., Rao F., Song S. (2017). Reexamining Calcination of Kaolinite for the Synthesis of Metakaolin Geopolymers—Roles of Dehydroxylation and Recrystallization. J. Non-Cryst. Solids.

[20] Gharzouni A. (2016). Contrôle de l’attaque Des Sources Aluminosilicates Par La Compréhension Des Solutions Alcalines. Ph.D. Thesis.

[21] Pouhet R., Cyr M. (2016). Formulation and Performance of Flash Metakaolin Geopolymer Concretes. Constr. Build. Mater..

[22] San Nicolas R., Cyr M., Escadeillas G. (2013). Characteristics and Applications of Flash Metakaolins. Appl. Clay Sci..

[23] Almusallam T. (2014). Effect of Sodium Silicate to Sodium Hydroxide Ratios on Strength and Microstructure of Fly Ash Geopolymer Binder. Chem. Eng..

[24] Phoo-ngernkham T., Maegawa A., Mishima N., Hatanaka S., Chindaprasirt P. (2015). Effects of Sodium Hydroxide and Sodium Silicate Solutions on Compressive and Shear Bond Strengths of FA—GBFS Geopolymer. Constr. Build. Mater..

[25] Matalkah F., Aqel R., Ababneh A. (2020). ScienceDirect ScienceDirect ScienceDirect Enhancement of the Mechanical Properties of Kaolin Geopolymer Enhancement of Sodium the Mechanical Properties of Kaolin Geopolymer Using Hydroxide and Calcium Oxide Using Sodium Hydroxide and Calcium Oxide. Procedia Manuf..

[26] Matalkah F., Xu L., Wu W., Soroushian P. (2017). Mechanochemical Synthesis of One-Part Alkali Aluminosilicate Hydraulic Cement. Mater. Struct./Mater. Et Constr..

[27] Bouchenafa O., Hamzaoui R., Bennabi A., Colin J. (2019). PCA Effect on Structure of Fly Ashes and Slag Obtained by Mechanosynthesis. Applications: Mechanical Performance of Substituted Paste CEMI + 50% Slag/or Fly Ashes. Constr. Build. Mater..

[28] Kopp Alves A., Bergmann C.P., Berutti F.A. (2013). High-Energy Milling. In Novel Synthesis and Characterization of Nanostructured Materials.

[29] Bouchenafa O., Hamzaoui R., Azem L., Bennabi A., Colin J. Manufacturing Equivalent Clinker by Indirect Mechanosynthesis Process. Proceedings of the 1st International Conference on Innovations in Low-Carbon Cement & Concrete Technology.

[30] Rossana B., Carlo C., Paola M., Giovanna Z. (2009). Rocks with asbestos: Risk evaluation by means of an abrasion test. Am. J. Environ. Sci..

[31] Hamzaoui R., Bouchenafa O., Ben Maaouia O., Guessasma S. (2019). Introduction of Milled Kaolinite Obtained by Mechanosynthesis to Cement Mixture for the Production of Mortar: Study of Mechanical Performance of Modified Mortar. Powder Technol..

[32] Yaseri S., Hajiaghaei G., Mohammadi F., Mahdikhani M., Farokhzad R. (2017). The Role of Synthesis Parameters on the Workability, Setting and Strength Properties of Binary Binder Based Geopolymer Paste. Constr. Build. Mater..

[33] Rożek P., Król M., Mozgawa W. (2018). Spectroscopic Studies of Fly Ash-Based Geopolymers. Spectrochim. Acta—Part A Mol. Biomol. Spectrosc..

[34] Wu F., Li H. (2021). Effects Of High Salinity Wastewater On The Properties Of Coal Gasification Residue-Based Cementitious Material. Therm. Sci..

[35] Rees C.A., Provis J.L., Lukey G.C., Van Deventer J.S.J. (2007). In Situ ATR-FTIR Study of the Early Stages of Fly Ash Geopolymer Gel Formation. Langmuir.

[36] Allali F., Joussein E., Kandri N.I., Rossignol S. (2016). The Influence of Calcium Content on the Performance of Metakaolin-Based Geomaterials Applied in Mortars Restoration. Mater. Des..

[37] Loy C.W., Matori K.A., Lim W.F., Schmid S., Zainuddin N., Wahab Z.A., Alassan Z.N., Zaid M.H.M. (2016). Effects of Calcination on the Crystallography and Nonbiogenic Aragonite Formation of Ark Clam Shell under Ambient Condition. Adv. Mater. Sci. Eng..

[38] Yousuf M., Mollah A., Hess T.R., Tsai Y.N., Cocke D.L. (1993). An FTIR and XPS Investigations of the Effects of Carbonation on the Solidification/Stabilization of Cement Based Systems-Portland Type V with Zinc. Cem. Concr. Res..

[39] Alehyen S., El Achouri M., Taibi M. (2017). Characterization, Microstructure and Properties of Fly Ash-Based Geopolymer. J. Mater. Environ. Sci..

[40] Davidovits J. (2020). Geopolymer Chemistry and Applications.

[41] Mobili A., Tittarelli F. (2020). Applied Sciences One-Part Alkali-Activated Pastes and Mortars Prepared with Metakaolin and Biomass Ash. Appl. Sci..

[42] Gharzouni A., Joussein E., Samet B., Baklouti S., Rossignol S. (2015). Effect of the Reactivity of Alkaline Solution and Metakaolin on Geopolymer Formation. J. Non-Cryst. Solids.

[43] Kassem N.N., Kishar E.A., Ahmed D.A. (2021). Effect of Elevated Temperatures on The Performance of Metakaolin Geopolymer Pastes Incorporated by Cement Kiln Dust. Egypt. J. Chem..

[44] Tantawy M.A. (2017). Effect of High Temperatures on the Microstructure of Cement Paste. J. Mater. Sci. Chem. Eng..

[45] Nikolov A., Rostovsky I., Nugteren H. (2017). Geopolymer Materials Based on Natural Zeolite. Case Stud. Constr. Mater..

[46] Yip C.K., Van Deventer J.S.J. (2003). Microanalysis of Calcium Silicate Hydrate Gel Formed within a Geopolymeric Binder. J. Mater. Sci..

[47] Chindaprasirt P., Silva P. (2012). De Effect of SiO_2_ and Al_2_O_3_ on the Setting and Hardening of High Calcium Fly Ash-Based Geopolymer Systems. J. Mater. Sci..

[48] Feng Y., Kero J., Yang Q., Chen Q., Engström F. (2019). Mechanical Activation of Granulated Copper Slag and Its Influence on Hydration Heat and Compressive Strength of Blended Cement. Materials.

[49] Paul D.K., Gnanendran C.T. (2016). Characterization of lightly stabilized granular base materials using monotonic and cyclic load flexural testing. J. Mater. Civ. Eng..

[50] Mohsen A., Kohail M., Alharbi Y.R., Abadel A.A., Soliman A.M., Ramadan M. (2023). Case Studies in Construction Materials Bio-Mechanical Efficacy for Slag/Fly Ash-Based Geopolymer Mingled with Mesoporous NiO. Case Stud. Constr. Mater..

[51] Pol I., Tero S., Juho L., Harisankar Y., Anne S., Damø J. (2022). Comparison of One—Part and Two—Part Alkali—Activated Metakaolin and Blast Furnace Slag. J. Sustain. Metall..

[52] Nath S.K. (2018). Geopolymerization Behavior of Ferrochrome Slag and Fly Ash Blends. Constr. Build. Mater..

[53] Gharzouni A., Samet B., Baklouti S., Joussein E., Rossignol S. (2016). Addition of Low Reactive Clay into Metakaolin-Based Geopolymer Formulation: Synthesis, Existence Domains and Properties. Powder Technol..

[54] Yip C.K., Lukey G.C., Van Deventer J.S.J. (2005). The Coexistence of Geopolymeric Gel and Calcium Silicate Hydrate at the Early Stage of Alkaline Activation. Cem. Concr. Res..

[55] San R., Approche N. (2012). Approche Performantielle Des b ´Etons Avec m´ Etakaolins Obtenus Par Calcination Flash Rackel San Nicolas. https://theses.hal.science/tel-00756481v1/document.

[56] Lizcano M., Kim H.S., Basu S., Radovic M. (2012). Mechanical Properties of Sodium and Potassium Activated Metakaolin-Based Geopolymers. J. Mater. Sci..

[57] Xu H., Van Deventer J.S.J. (2000). The Geopolymerisation of Alumino-Silicate Minerals. Int. J. Miner. Process..

